# Unmasking the Hidden Morbidity of Ocular Diseases in Primary Care Through a Collaboration with Specialists in Remote Areas: A Cross-Sectional Study from Rural Crete, Greece

**DOI:** 10.3390/diseases13050137

**Published:** 2025-04-29

**Authors:** Konstantinos Chliveros, Manolis Linardakis, Ioanna Tsiligianni, Miltiadis Tsilimbaris, Ioannis Pallikaris, Christos Lionis

**Affiliations:** 1 Clinic of Social and Family Medicine, Faculty of Medicine, University of Crete, 71500 Crete, Greece; linman@med.uoc.gr (M.L.); i.tsiligianni@uoc.gr (I.T.); lionis@galinos.med.uoc.gr (C.L.); 2 Ophthalmology Clinic, University Hospital of Heraklion, 71500 Crete, Greece; tsilimb@med.uoc.gr (M.T.); pallikar@med.uoc.gr (I.P.)

**Keywords:** epidemiological study, eye diseases, hidden morbidity, primary care, rural and remote

## Abstract

**Background**: Ocular disorders are not frequently addressed in primary care, which is more visible in remote rural settings. The aim of the study was to assess the prevalence and pattern of eye diseases in a remote rural population of Crete and to explore whether they represent a hidden morbidity. **Materials and Methods**: A community-based, cross-sectional study based on data collected through a comprehensive clinical investigation conducted by a mobile ophthalmological unit. Permanent inhabitants, aged over 40 years, living in one remote rural community located on the highest mountain of Crete, were invited to participate. The prevalence of eye diseases was measured during the comprehensive ophthalmological examination. Patients’ medical records were used to assess hidden morbidity. The National Eye Institute Visual Function Questionnaire-25 (NEI VFQ-25) was applied to measure self-reported vision-targeted health status. **Results**: A total of 239 individuals agreed to participate; 54.9% were females (*n* = 151), with a mean ageof 66.13 years (±14.56). The most common diagnoses were refractory errors (59%), cataract (21.7%), glaucoma (11.7%), maculopathy (8.8%), and dry eyes (8.8%). A previously undiagnosed eye disorder was detected in 34.3% (*n* = 82). Total scores of NEI VFQ-25 measured quality of life were highand significantly lower in Known Cases of eye diseases compared to patients with New or Without diagnosis (76.6 vs. 84.1 and 84.6, respectively, *p* = 0.009). **Conclusions**: Our study highlighted the need for increased awareness of primary care in rural areas concerning eye disorders. Local policies should focus on implementing public health interventions and encouraging close cooperation with specialists to overcome accessibility issues.

## 1. Introduction

The declarations of the World Health Organization (WHO), such as Alma-Ata in 1978 and Astana in 2018, have long recognized the vital role of Primary Health Care (PHC) in reducing inequalities and tackling social isolation, particularly in underserved and geographically isolated areas. Both declarations underscore the need for comprehensive, person-centered care through the strengthening of PHC systems to achieve universal health coverage and promote health equity globally [1].

Primary care, essential and crucial for rural areas, encounters various challenges such as socio-economic deprivation, a diminishing and aging population, geographic isolation, and lack of important infrastructure, all of which impede diagnosis, treatment, and follow-up [2]. Moreover, specialized care services are of limited access for rural communities, as most specialists practice in urban areas, and purchasing the necessary specialized equipment is proven challenging in a rural primary care setting [3]. Predisposing and personal features like age, gender, marital status, education level, occupation, household income, self-perceived quality of life, family history of eye disease, chronic conditions, and rural versus urban residence have been identified as factors influencing the utilization of eye care services [4,5]. Particularly in countries with numerous rural and remote areas, there is a clear need to bring specialists to efficient collaboration with primary care [6].

Visual Impairment (VI) negatively impacts every aspect of an individual’s life [7]. Worldwide, age-related eye diseases such as cataract, macular degeneration, glaucoma, and diabetic retinopathy are the primary causes of visual impairment and blindness [8,9,10]. Ocular diseases exhibit an increased tendency to occur around the age of 40, with a notable rise after the age of 60 [11,12]. Despite being treatable and preventable, the affected population is anticipated to experience a significant increase in the coming years, particularly in the post-COVID-19 period [13,14]. The growing aging population is anticipated to worsen the problem, particularly in low- and middle-income countries, as well as in rural areas [4,15]. According to WHO reports, the optimal strategy for reducing avoidable blindness involves bringing eye healthcare services to people in rural communities through outreach programs [16]. This approach is crucial since the prevalence of blindness and visual impairment is higher among rural inhabitants compared to urban populations [17].

In Greece, after a long period of the financial crisis and austerity measures, hypertension and cardiovascular diseases, followed by respiratory diseases, rank among the most prevalent chronic conditions with progressive deterioration [18,19]. Ocular diseases are also common in the Greek general population, although often neglected, with limited available data [20]. The aging of the population, as well as the advancements in medical care, contribute to an increasing number of individuals facing one or multiple chronic conditions [21]. A high prevalence of multimorbidity indicates higher mortality, large and growing rural-urban and within-rural mortality, and increased healthcare utilization [22]. Patients with visual impairment and concomitant comorbidities seem to be more likely to seek eye care [23]. The impact of ocular diseases on quality of life (QoL) has been thoroughly documented in a series of studies, employing various instruments to evaluate patients’ subjective perceptions of their health [24,25]. Nevertheless, studies focusing on vision-specific quality of life (VS-QoL) in Greek populations are limited [26,27]. Visual impairment is a significant public health concern as it not only diminishes the quality of life by disrupting daily activities and increasing morbidity and mortality but also imposes a substantial financial burden, contributing to the rapid expansion of healthcare expenditures [23,28].

The island of Crete in Greece, as the most southern part of Europe, is world famous for its sunny beaches. What is less known is that, in fact, it is a rather mountainous island with plenty of rural and isolated communities. Primary care in Greece is structured partially as a branch of the National Health System, offered free of charge for all citizens [29]. Additionally, there is a growing presence of private healthcare services [30]. Despite multiple healthcare reform efforts, healthcare services are less developed in remote and rural communities, and primary care is mainly delivered either by general practitioners (GPs) employed in primary care centers and regional surgeries or by rural unspecialized physicians [31]. Ocular care and other specialized forms of care are typically available in urban private care or hospital settings [32]. Therefore, it was of special interest to explore to what extent the ocular healthcare needs of a rural community on the island of Crete are well recognized or diagnosed by the local primary care services. The primary objectives of this cross-sectional study were to assess the burden of ocular diseases and visual impairment and to reveal the hidden morbidity within this rural population. Moreover, to assess the impact of local demographics and specific social and cultural characteristics on the measured prevalence of ocular diseases and the related patients’ attitudes [33].

## 2. Materials and Methods

### 2.1. Study Design and Setting

A community-based, cross-sectional study was based on data from a comprehensive clinical investigation conducted by a mobile ophthalmological unit in Crete. The mainland in Crete is mostly mountainous, with over half of the population living in more than 800 small villages, most of them rural and remote. In our study of a small and well-defined remote rural community of approximately 1117 inhabitants (according to the 2021 National Census), located on the highest mountain of Crete, primary care services are offered by two GPs working together in a regional surgery. The nearest primary care center is in a larger village, approximately 7 km away. However, specialized eye care is only accessible in the capital city of Heraklion, 43 km away, with the road network and basic infrastructure often damaged or in poor condition, challenging the accessibility of this remote rural setting.

### 2.2. Participants

All permanent inhabitants aged over 40 years were invited to participate in the study, providing their written consent. The population presents unique cultural and anthropological characteristics, is mostly indigenous, and is relatively genetically preserved.

### 2.3. Data Collection and Tools

Patients’ demographic and lifestyle data and medical history were obtained through interviews. The examination variables incorporated socio-demographic characteristics (age, gender, education, family status, occupation), lifestyle habits, diagnoses of common chronic diseases, and eye disorder diagnoses (both new and old cases) coded using the International Classification of Diseases, Tenth Revision(ICD-10). In addition to the comprehensive ophthalmological examination conducted with portable yet high-tech equipment [34], we utilized the 25-item version of the National Eye Institute Visual Function Questionnaire (NEI VFQ-25) [35]. The NEI VFQ-25, a tailored 25-item version of the National Eye Institute Visual Function Questionnaire, was specifically designed for individuals dealing with common chronic eye conditions associated with aging. These conditions include cataract, diabetic retinopathy, age-related maculopathy, and glaucoma. It serves as a well-documented tool that associates ocular disorders with health-related quality of life, as well as with common chronic medical conditions of adulthood, e.g., depression. The NEI VFQ-25 consists of 25 vision-targeted questions representing eleven vision-related subscales, as follows: General Health, General Vision, Ocular pain, Near activities, Distance activities, Vision-Specific Social Functioning-Mental Health-Role Difficulties-Dependency, Color Vision, and Peripheral Vision. All items are scored so that higher scores represent better functioning. Each item is then converted to a 0 to 100 scale so that the lowest and highest possible scores are set at 0 and 100 points, respectively. An overall composite score is also calculated by averaging calculated subscale scores [25,35,36]. The Greek standardized version of the National Eye Institute Visual Function Questionnaire (NEI VFQ-25), produced by the Laboratory of Experimental Ophthalmology at Aristotle University of Thessaloniki, Greece, was administered and completed [36]. One ophthalmology team from the University Hospital of Heraklion collected ophthalmological data, including visual acuity, intraocular pressure, and funduscopy. The comprehensive ophthalmological examination involved measuring ocular acuity using a Snellen chart, autorefractometry, slit lamp biomicroscopy, tonometry, and fundus examination post-dilation. Specific eye diseases covered in the study included conjunctivitis, lacrimation, pterygium, cataract, glaucoma, macular degeneration, and retinal detachment. The primary goals of the study included determining the prevalence of common eye disorders, adjusting for socio-demographic factors, assessing their severity in terms of impaired quality of life, and the need for further investigation.

### 2.4. Ethics Approval

All procedures performed in studies involving human participants were in accordance with the ethical standards of the Ethics Committee of the University of Crete. Special attention was given to confidentiality and anonymity regarding the data collected, following the principles of the 1964 Helsinki Declaration of Human Rights and its later amendments or comparable ethical standards. Ethics approval was granted by the Health Research Ethics Committee of the University Hospital of Heraklion, Crete. Written and signed informed consent was obtained from all participants.

### 2.5. Statistical Analysis

The data were analyzed using the Statistical Package for the Social Sciences (IBM-SPSS, version 25, 2011, Chicago, IL, USA). Prevalence of chronic diseases and multimorbidity was estimated with corresponding 95% confidence intervals (95% CIs). Gender differences in descriptive characteristics were estimated by Chi-square (χ^2^) and Student *t*-tests, and in frequencies of various risk factors or mean levels of different scales by Chi-square (χ^2^) and Mann–Whitney tests. The symmetry of the NEI VFQ-25 scale and subscale levels was assessed with Blom’s test (QQ plot). Finally, Kruskal–Wallis nonparametric tests were performed on the NEI VFQ-25 scale and subscales in relation to diagnosed cases with ocular symptoms/diseases.

## 3. Results

### 3.1. Demographics

The basic demographic characteristics of the 239 individuals who agreed to participate and provided data for full analysis (the total population in this community aged >40 years was 255 people) are shown in Table 1. 54.9% were females (*n* = 133) and 44.4% were males (*n* = 106), with the mean age of all being 66.4 years (±14.8). 47.3% were uneducated, while the majority were married (78.7%) or had 3 or more children (71.5%). Regarding occupational status, 56.1% were retired/housewives, while 29.9% were farmers or livestock farmers. It was estimated that statistically more females than males were uneducated (58.6% vs. 33.0%, *p* < 0.05) or lived alone, meaning they were unmarried, divorced, or widowed (32.3% vs. 7.5%, *p* < 0.05) (χ^2^ and Student *t* tests between males and females).

**Figure 1:**
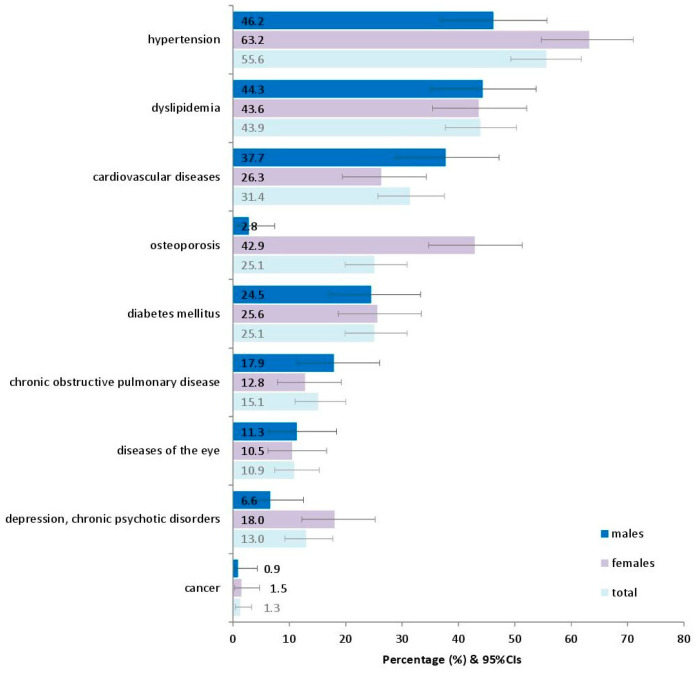
Prevalence of various reported chronic conditions in 239 adults in the current study. **Footnote of Figure 1:** Diseases of the eye include conjunctivitis, lacrimation, pterygium, cataract, glaucoma, macular degeneration, and retinal detachment, as reported in the patients’ medical history. 95%CIs: 95% confidence intervals.

Figure 1 presents the prevalence of various chronic conditions in adults as they have been recorded in the interviews with the participants. The most prevalent seemed to be hypertension (55.6%), followed by dyslipidemia (43.9%) and cardiovascular diseases (31.4%).

### 3.2. The Most Frequent Diagnoses

The most frequently diagnosed eye diseases were refractive errors (myopia, astigmatism, presbyopia, hyperopia) (*n* = 141; 59.0%), cataract (*n* = 52; 21.8%), glaucoma (*n* = 28; 11.7%) (Table 2), maculopathy (*n* = 21; 8.8%), dry eyes (*n* = 21; 8.8%), pterygium (*n* = 10; 4.2%), and diabetic retinopathy (*n* = 10; 4.2%). Among the women diagnosed with eye diseases, 66% had refractive errors, 19.5% had cataract, 14.3% had glaucoma, and 48.9% had more than three chronic conditions. Similarly, 50% of men were diagnosed with refractive errors, 24.5% with cataract, 8.5% with glaucoma, and 39.6% with other chronic conditions. The prevalence of eye diseases was higher with age, with >70% of those over 60 being diagnosed with refractive errors. Cataract and glaucoma were also often found, especially in the elderly aged >80 years (33.3%and 20.8%, respectively). In relation to educational status, people with no education were mainly diagnosed with refractive errors (69.9%), while 33.6% were diagnosed with cataract, and 10.6% had glaucoma. Adults living alone were diagnosed with more refractive errors (72.5%) and cataract (29.4%) compared to married individuals (55.3% and 19.7%), while for glaucoma, it was married individuals who were diagnosed more often than single, divorced, or widowed people (29.4% versus 19.7%). Regarding occupation, the prevalence of refractive errors was 73.1% for retired/household, 42% for public sector workers and self-employed, and 58.5% for farmers and livestock farmers. According to a chi-square test analysis of the most prevalent chronic conditions in relation to eye diseases, refractive errors were observed with significantly higher frequency in participants with hypertension (65.4% vs. 50.9%, *p* = 0.024), dyslipidemia (78.1% vs. 44%, *p* < 0.001), and cardiovascular diseases (CVDs) (75.7% vs. 51.8%, *p* < 0.001). Cataract also showed significant correlations to hypertension (*p* < 0.001) and CVDs (*p* < 0.001), while glaucoma was more common in participants with diabetes (20% vs. 8.9%, *p* = 0.021) (Table 2).

### 3.3. Hidden Morbidity

**Figure 2:**
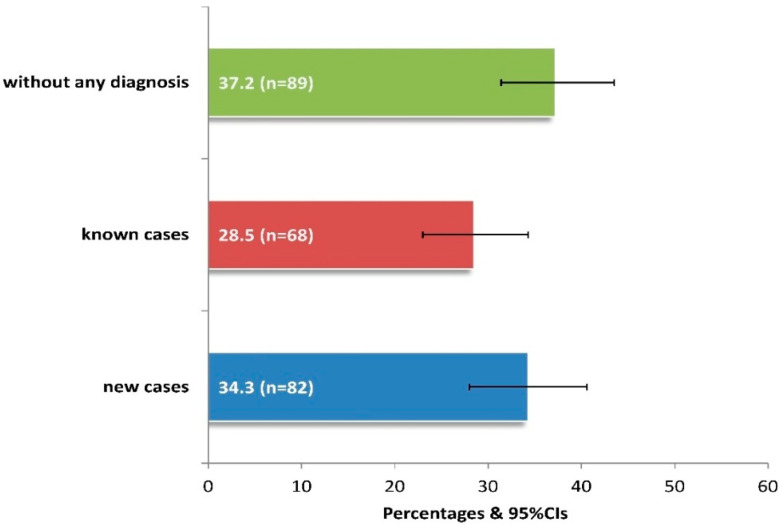
Prevalence of newly diagnosed cases with clinically observed ocular symptoms-diseases in 239 adults of the current study. **Footnote of Figure 2:** The “known cases” include 23 cases of new diagnoses of a different disease from the existing one, as observed by clinical examination. 95%CIs: 95% confidence intervals.

After a thorough ophthalmological examination, 62.8% of patients presented ocular symptoms/diseases, 34.3% being diagnosed for the first time (Figure 2). Among those unaware of having eye disease, it was estimated that significantly more patients aged between 60 and 79 years versus those aged 40–59 or >80 years had a new diagnosis (64.6% vs. 15.9% or 19.5%, *p* < 0.05), while those without education also had significantly more new diagnoses compared to the rest of the sample (*p* < 0.001).

Similarly, public sector workers and self-employed people suffering from more than 3 chronic conditions (*p* < 0.001) were more likely not to know that they had an eye disease (Table 3).

Overall, 43.9% (*n* = 105) of patients, including those with Known disease, were referred to the University Hospital for further assessment, mainly in order to receive proper treatment (10.5%), for a complete funduscopy (7.6%), and for follow-up (6.7%), especially those diagnosed with glaucoma.

### 3.4. National Eye Institute Visual Function Questionnaire (NEI VFQ-25) Scoring

According to NEI VFQ-25 responses in relation to diagnosed cases with ocular symptoms/diseases, the measured scores were considerably high for the 239 participants, as the scale is assessed with score limits from 0 to 100, where 100 is defined as better functional health. However, multivariate analysis revealed significantly lower scores, calculating the total score of the Vision Functioning Scale, for the group of Known Cases in relation to New or Without diagnosis (71.5 vs. 81.9 and 90.6, respectively, *p* < 0.001). Analyzing further the scores of every NEI VFQ-25 subscale, the group of participants with Known Cases compared to New or Without diagnosis reported significantly lower levels of health for all subscales, measured as General Health, General Vision, Ocular Pain, Near Activities, Distance Activities, Vision-specific Social Functioning, Vision-specific Mental Health, Vision-specific Role Difficulties, Vision-specific Dependency, Color Vision, and Peripheral Vision (*p* < 0.05) (Table 4).

## 4. Discussion

### 4.1. Main Findings

Ocular problems were highly prevalent in our isolated rural community in the mountains of Crete. This is in accordance with another study conducted in Cretan villages, where the most frequent diseases were cataract (24.8%), refractive errors (9.5%), glaucoma (5.4%), and macular hole (3.0%), with women and the elderly (age group > 60 years) presenting higher risk, especially of glaucoma and cataract [20]. In the current study, no significant difference in prevalence was observed between males and females, while increased age was accompanied by significant eye morbidity. The fact that the study population was over 40 years of age partially explains the high prevalence of ocular diseases, since aging is strongly related to both cataract and glaucoma [11,12].

### 4.2. Discussion of the Study Findings in Light of the Literature

The high prevalence of cataract in the present study is in concordance with similar studies. The Indian Council of Medical Research (ICMR) collaborative study showed variations in the prevalence of cataract ranging from 30.1% to 72.2% in different parts of India [37]. In a cross-sectional study in rural Malaysia, visual impairment was noted in 8.2% of patients, with cataract accountingfor the most common eye disease (22.9%), responsible for severe visual impairment, followed by retinal diseases (11.5%) and ocular trauma (9.8%) [38]. In 2020, according to a systematic review, the leading global causes of blindness in those aged 50 years and older were cataract (15.2 million cases), followed by glaucoma (3.6 million cases) [8]. The elevated prevalence of cataract in the present study aligns with findings from other studies. A population-based cross-sectional study by the Department of Ophthalmology and Department of Community Medicine in Northern India showed that the most common causes of blindness were cataract, followed by corneal opacity, glaucoma, refractive error, diabetic retinopathy, age-related macular degeneration, etc., highlighting that ocular morbidity tends to increase around the age of 40, with a steep increase after the age of 60 [12]. A systematic scoping review in South Africa also showed that the leading causes of blindness were untreated cataracts (54%), glaucoma (17%), and diabetic retinopathy (57%) [10]. Also, a recent meta-analysis in China found that visual impairment causes a great health burden among Chinese populations, particularly affecting females, people dwelling in rural areas, older and those with lower educational levels [9]. The fact that eye disorders represent hidden morbidity has been observed in another study where a large proportion of the individuals who reported having vision problems or wearing glasses did not receive quality vision care [39]. Almost half (43.9%) of the screened patients required follow-up care, thus indicating the high prevalence of unmet eye care in this population. This referral rate is comparable to that of other screening initiatives with similar referral criteria [40]. A living rural community is dependent on the health of its population. Access to medical care does not guarantee good health [40]. However, access to healthcare is critical for a population’s well-being and optimal health. People living in urban areas are more likely to visit ophthalmologists compared with people living in rural areas [2,3,4]. Barriers to healthcare result in unmet healthcare needs, including a lack of preventive and screening services, treatment of illnesses, and preventing patients from needing costly hospital care [7,11,12,33]. According to the comorbidity data, particular chronic health conditions were found to coexist with common visual diseases. Similar strong associations were also shown in other studies, especially for hypertension and cardiovascular disorders, indicating a higher prevalence of cardiovascular chronic conditions among those with visual impairment compared with those without visual impairment [23]. Vision-Related Quality of Life (VRQoL) was found to be affected, especially mental health issues, and for known cases, according to NEI VFQ-25 scores. Older adults with chronic medical conditions, including eye disease and vision impairment, were found to be prone to depression. Depression is known to have a strong, significant impact on health-related quality of life, both associated with a reduced NEI VFQ-25 total score and a reduced score in the subscale of mental health in another study on older adults [25]. According to a systematic review that examined the association between vision impairment or eye disease and quality of life, vision impairment and eye diseases, namely glaucoma, diabetic retinopathy, and age-related macular degeneration, were associated with lower quality of life, using a range of outcome measures, including NEI VFQ-25 [24]. In a Greek population, the vision-related QoL of patients with glaucoma has been measured using the NEI VFQ-25, confirming the impact of visual field defects on VS-QoL, as strong correlations were found between self-reported visual deterioration and quantified visual dysfunction [26]. Many studies have explored associations between ocular disorders and survival. Overall, individuals with ocular disorders have been demonstrated to have an elevated risk of mortality compared to those without such disorders. These associations have been shown with cataract, glaucoma, diabetic retinopathy, visual impairment, and recently with age-related maculopathy (ARM) [22,23,28].

### 4.3. Study Limitations and Strengths

The main limitation of our study is the small sample size, coming from one remote rural community in the mountains of Crete, making our findings not easily generalized to a larger population. Despite this limitation, our findings on the prevalence of ocular diseases (refractive errors, cataract, glaucoma) were similar to those of larger studies in rural areas [8,10,12]. Depression, as being high in the elderly, could have influenced their self-perception of the burden of eye disease, which could have affected the measured vision-related QoL, reflected in low NEI VFQ-25 scores for mental health in our population [25]. The high mean age and the low educational level of the participants could explain the low scores in the visual function scale found in the current study. These findings support the hypothesis that the onset of severe visual impairment might be a causal factor for the decline in physical and cognitive function, extending our previous knowledge of an association between these factors [24,25,26,27,28]. Our study, however, was performed in a rural and remote area, where opportunities to conduct research and provide specialized care are limited. Mobile specialized services have been used for the first time, showing that organized and integrated care is feasible and vital for those communities. Regarding the design of the study, the inclusion of a highly equipped, mobile, specialized ophthalmological team, which provides easy access to advanced eye examinations at a university hospital, was assumed as a significant plus. The local population’s high participation underlined the importance of such initiatives when primary and secondary/tertiary care collaborate in a timely, effective, and person-oriented manner to improve the health and well-being of the whole community [5].

### 4.4. Implications

This cross-sectional study has several implications, primarily for clinical management, but also for education and health policies. The ratio of newly diagnosed cases to already known cases was high, with cataract, maculopathy, and glaucoma presenting the highest ratio. Since maculopathy and glaucoma both progress silently for a long period before they induce symptoms, often resulting in severe visual impairment, people are less likely to seek eye care early in the course of the disease [15,16,17]. Therefore, primary care priorities should include systematic screening for ocular diseases, especially in remote and rural areas, where access to specialized units is restricted [3,6]. The challenges that rural residents face in accessing healthcare services contribute to health disparities [2]. Rural risk factors for health disparities include geographic isolation, lower socio-economic status, and higher rates of health risk behaviors [4,5]. Rural residents may not get preventive screening that can lead to early detection and treatment of disease [7,11,12]. A well-trained rural physician is crucial in addressing current challenges, and it is true that they require only minimal diagnostic equipment to conduct ocular screenings. Gaining insights from our study would underscore the importance of social determinants of health in terms of developing and implementing public health interventions to improve health care in isolated rural communities [20]. Our results reveal the important issue of hidden morbidity for ocular diseases among the rural population, a warning finding of great importance for primary care providers in similar rural areas of Southern Europe [4]. Higher rates of chronic illness and poor overall health are found in rural communities compared to urban populations [21,22,23]. The role of GPs providing care to the community needs to be expanded to include efficient management of ocular diseases in close collaboration with specialist eye teams [41]. The COVID-19 pandemic revealed the need to empower primary care to manage the tremendous physical and mental effects on individuals and communities [3,13,14]. Although many policies and programs are run by local authorities, the success and effectiveness of these programs are questionable due to gaps in their implementation. A mobile, highly equipped ophthalmological unit could represent an excellent solution for remote areas, where the available resources cannot handle the level of demand for eye care [3,42].

## 5. Conclusions

Overall, reducing visual impairment, enhancing preventive eye care, and promoting the utilization of vision rehabilitation services emerge as crucial public health priorities. Despite improvements in healthcare accessibility accomplished by a series of medical and healthcare reforms, disparities remain, especially between urban and rural areas. Quality health care is largely unaffordable and inaccessible to populations from lower socio-economic strata residing in remote areas. Our study presents the high impact of ocular diseases in remote rural areas, suggests strategies to overcome accessibility issues, and promotes early detection. Future larger studies hold the potential to provide more comprehensive data, aiding policymakers in effective planning. The insights gained from such studies can contribute to informed decision-making and the development of targeted policies to address healthcare needs in remote rural areas.

## Figures and Tables

**Table 1:** Characteristics of 239 adults aged 40–100 years.

*Characteristics*		n	%
Gender	Males	106	44.4
	Females	133	55.6
Age, *years*	Mean ± Stand. dev. [min, max]	66.4 ± 14.8 [40, 100]	
	40–59	73	30.5
	60–79	118	49.4
	80+	48	20.1
Education	None	113	47.3
	Primary school	82	34.2
	High school	41	17.2
	University	3	1.3
Family status	Unmarried, divorced, widows	51	21.3
	Married	188	78.7
Number of offspring	None	16	6.7
	1	14	5.9
	2	38	15.9
	3+	171	71.5
Occupation	Unemployed	15	6.3
	Retired, household	134	56.1
	Public sector worker, self-employed, etc.	19	7.9
	Livestock farmer	63	26.4
	Farmer	8	3.3
Chronic conditions	3+	107	44.8
Diseases of the eye	yes	26	10.9

**Table 2:** Diagnosed cases of eye diseases and comorbidity (chronic conditions) in 239 adults aged 40–100 years of the study in relation to their characteristics.

Characteristics	Refractive Errors ^a^		Cataract		Glaucoma		Hypertension		Dyslipidemia		Cardiovascular Diseases		Osteoporosis		Diabetes Mellitus		Chronic Obstructive Pulmonary Disease		Diseases of the Eye		Depression-Chronic Psychotic Disorders		Cancer		Dementia		Asthma		Parkinson’s Disease		Chronic Conditions (3+) ^b^	
	n	%	n	%	n	%	n	%	n	%	n	%	n	%	n	%	n	%	n	%	n	%	n	%	n	%	n	%	n	%	n	%
Number	141	59.0	52	21.8	28	11.7	133	55.6	105	43.9	75	31.4	60	25.1	60	25.1	36	15.1	26	10.9	31	13.0	3	1.3	2	0.8	1	0.4	1	0.4	107	44.8
Gender																																
Males	53	50.0	26	24.5	9	8.5	49	46.2	47	44.3	40	37.7	3	2.8	26	24.5	19	17.9	12	11.3	7	6.6	1	0.9	2	1.9	1	0.9	1	0.9	42	39.6
Females	88	66.2	26	19.5	19	14.3	84	63.2	58	43.6	35	26.3	57	42.9	34	25.6	17	12.8	14	10.5	24	18.0	2	1.5	-		-		-		65	48.9
Age, years																																
40–59	22	30.1	2	2.7	4	5.5	15	20.5	21	28.8	5	6.8	5	6.8	10	13.7	1	1.4	4	5.5	11	15.1	-		-		-	1.4	-		8	11.0
60–79	83	70.3	34	28.8	14	11.9	78	66.1	65	55.1	43	36.4	33	28.0	32	27.1	25	21.2	13	11.0	13	11.0	3	2.5	-		-		-		66	55.9
80+	36	75.0	16	33.3	10	20.8	40	83.3	19	39.6	27	56.3	22	45.8	18	37.5	10	20.8	9	18.8	7	14.6	-		2	4.2	-		1	2.1	33	68.8
Education																																
None	79	69.9	38	33.6	12	10.6	78	69.0	52	46.0	44	38.9	47	41.6	32	28.3	20	17.7	16	14.2	15	13.3	3	2.7	1	0.9	-		-		63	55.8
Primary School	46	56.1	13	15.9	13	15.9	44	53.7	37	45.1	23	28.0	13	15.9	23	28.0	15	18.3	7	8.5	8	9.8	-		1	1.2	1	1.2	1	1.2	36	43.9
High School	15	36.6	1	2.4	3	7.3	11	26.8	16	39.0	7	17.1	-		5	12.2	1	2.4	2	4.9	8	19.5	-		-		-		-		8	19.5
University	1	33.3	-		-		-		-		1	33.3	-		-		-		1	33.3	-		-		-		-		-		-	
Family status																																
Unmarried, Divorced, Widowed	37	72.5	15	29.4	3	5.9	34	66.7	24	47.1	20	39.2	31	60.8	15	29.4	9	17.9	13	25.5	8	15.7	1	2.0	-		-		-		30	58.8
Married	104	55.3	37	19.7	25	13.3	99	52.7	81	43.1	55	29.3	29	15.4	45	23.9	27	14.4	13	6.9	23	12.2	2	1.1	2	1.1	1	0.5	1	0.5	77	41.0
Number of offsprings																																
none	9	56.3	4	25.0	3	18.8	8	50.0	3	18.8	7	43.8	6	37.5	3	18.8	4	25.0	2	12.5	2	12.5	1	6.3	1	6.3	-		1	6.3	8	50.0
1	8	57.1	5	35.7	-		7	5.0	3	21.4	6	42.9	-		2	14.3	2	14.3	2	14.3	1	7.1	-		-		-		-		4	28.6
2	19	50.0	9	23.7	4	10.5	17	44.7	18	47.4	5	13.2	10	26.3	7	18.4	8	21.1	6	15.8	5	13.2	-		-		-		-		13	34.2
3+	105	61.4	34	19.9	21	12.3	101	59.1	81	47.4	57	33.3	44	25.7	48	28.1	22	12.9	16	9.4	23	13.5	2	1.2	1	0.6	1	0.6	-		82	48.0
Occupation																																
Unemployed	5	33.3	-		3	20.0	3	20.0	4	26.7	-		1	6.7	3	20.0	1	6.7	-		3	20.0	-		-		-		-		4	26.7
Retired, Household	98	73.1	38	28.4	17	12.7	90	67.2	63	47.0	57	42.5	51	38.1	36	26.9	27	20.1	19	14.2	21	15.7	2	1.5	2	1.5	-		1	0.7	77	57.5
Public Sector Worker, Self-Employed, Etc.	8	42.1	1	5.3	2	10.5	5	26.3	5	26.3	5	26.3	-		2	10.5	1	5.3	3	15.8	4	21.1	-		-		-		-		3	15.8
Livestock Farmer	29	46.0	12	19.0	6	9.5	32	50.8	30	47.6	10	15.9	8	12.7	17	27.0	6	9.5	4	6.3	2	3.2	1	1.6	-		-		-		21	33.3
Farmer	1	12.5	1	12.5	-		3	37.5	3	37.5	3	37.5	-		2	25.0	1	12.5	-		1	12.5	-		-		1	12.5	-		2	25.0
Chronic conditions																																
3+	80	74.8	36	33.6	19	17.8	92	86.0	76	71.0	60	56.1	39	36.4	50	46.7	28	26.2	20	18.7	21	19.6	3	2.8	2	1.9	-		1	0.9	--	--

^a^ myopia, astigmatism, presbyopia, hyperopia. ^b^ 12 chronic conditions such as hypertension, dyslipidemia, cardiovascular diseases, osteoporosis, diabetes mellitus, chronic obstructive pulmonary disease, diseases of the eye, depression-chronic psychotic disorders, cancer, dementia, asthma, and Parkinson’s disease.

**Table 3:** Diagnosed cases with ocular symptoms—diseases in 239 adults aged 40–100 years of the study in relation to their characteristics.

Characteristics	Without Diagnosis	Known Cases	New Diagnosed Cases	*p*-Value
	n (%)			
Number	89 (37.2)	68 (28.5)	82 (34.3)	-
Gender				
Males	41 (46.1)	27 (39.7)	38 (46.3)	0.660
Females	48 (53.9)	41 (60.3)	44 (53.7)	
Age, years				
40–59	47 (52.8)	13 (19.1)	13 (15.9)	<0.001
60–79	34 (38.2)	31 (45.6)	53 (64.6)	
80+	8 (9.0)	24 (35.3)	16 (19.5)	
Education				
None	26 (29.2)	34 (50.0)	53 (64.6)	<0.001
Primary School	33 (37.1)	27 (39.7)	22 (26.8)	
High School	30 (33.7)	5 (7.4)	6 (7.3)	
University	-	2 (2.9)	1 (1.2)	
Family status				
Unmarried, Divorced, Widowed	15 (16.9)	18 (26.5)	18 (22.0)	0.341
Married	74 (83.1)	50 (73.5)	64 (78.0)	
Number of offsprings				
none	5 (5.6)	6 (8.8)	5 (6.1)	0.912
1	6 (6.7)	4 (5.9)	4 (4.9)	
2	17 (19.1)	9 (13.2)	12 (14.6)	
3+	61 (68.5)	49 (72.1)	61 (74.4)	
Occupation				
Unemployed	5 (5.6)	6 (8.8)	4 (4.9)	0.001
Retired, Household	38 (42.7)	47 (69.1)	49 (59.8)	
Public Sector Worker, Self-Employed, etc.	12 (13.5)	4 (5.9)	3 (3.7)	
Livestock Farmer	27 (30.3)	10 (14.7)	26 (31.7)	
Farmer	7 (7.9)	1 (1.5)	-	
Chronic conditions				
3+	17 (19.1)	46 (67.6)	44 (53.7)	<0.001

Chi-square tests (χ^2^).

**Table 4:** National Eye Institute Visual Function Questionnaire (NEI VFQ-25) in relation to diagnosed cases with ocular symptoms—diseases in 239 adults of the current study.

	Without Diagnosis (*n* = 89)	Known Cases (*n* = 68)	New Diagnosed Cases (*n* = 82)	
Scale and Subscales	Mean (Stand. Dev.)			*p*-Value
Visual Function Questionnaire (VFQ-25)	90.6 (17.6)	71.5 (26.3)	81.9 (17.4)	<0.001
General Health	68.8 (19.5)	49.1 (23.7)	54.1 (18.8)	<0.001
General Vision	74.6 (16.7)	56.3 (20.6)	67.3 (16.4)	<0.001
Ocular Pain	83.8 (19.5)	64.0 (26.9)	69.7 (27.9)	<0.001
Near Activities	91.0 (20.1)	70.8 (30.8)	81.3 (20.0)	<0.001
Distance Activities	93.5 (18.5)	70.7 (32.2)	83.1 (20.6)	<0.001
Vision-specific Social Functioning	95.4 (18.2)	81.0 (30.6)	89.3 (18.5)	<0.001
Vision-specific Mental Health	89.5 (18.9)	64.9 (30.8)	83.1 (19.8)	<0.001
Vision-specific Role Difficulties	93.7 (19.3)	74.2 (31.0)	87.0 (19.2)	<0.001
Vision-specific Dependency	95.6 (18.3)	79.0 (31.8)	91.5 (20.5)	<0.001
Color Vision	94.7 (19.4)	83.8 (29.9)	88.7 (21.2)	0.006
Peripheral Vision	94.4 (21.2)	71.3 (31.2)	79.0 (26.8)	<0.001

Kruskal–Wallis tests.

## Data Availability

Data are available from the corresponding author upon reasonable request due to restrictions, e.g., privacy, or ethical reasons.

## Abbreviations

The following abbreviations are used in this manuscript:

COVID-19	Corona Virus Disease 2019
CVD	Cardio Vascular Diseases
GPs	General Practitioners
ICD-10	International Classification of Diseases-Tenth Revision
LSD	Least Significant Difference
NEI VFQ-25	National Eye Institute Visual Function Questionnaire-25
PHC	Primary Healthcare
QoL	Quality of Life
VI	Visual Impairment
VRQoL	Vision-Related Quality of Life
VS-QoL	Vision-SpecificQuality of Life
WHO	World Health Organization
